# Microbial mechanisms in atherosclerosis: Revisiting the infectious hypothesis

**DOI:** 10.1016/j.athplu.2026.100602

**Published:** 2026-08-20

**Authors:** Carine El Hayek, Fredy Kahkedjian, Ghadir Amin, George W. Booz, Fouad A. Zouein

**Affiliations:** a The Cardiovascular, Renal, and Metabolic Diseases Research Center of Excellence, American University of Beirut Medical Center, Riad El-Solh, Beirut, Lebanon; b Department of Pharmacology and Toxicology, School of Medicine, University of Mississippi Medical Center, Jackson, MS, USA; c Department of Pharmacology and Toxicology, American University of Beirut Faculty of Medicine, Beirut, Lebanon

**Keywords:** Atherosclerosis, Plaque destabilization, Intraplaque biofilm, Biofilm dispersion, Planktonic transition, Vascular remodeling, Plaque rupture

## Abstract

Atherosclerosis is traditionally viewed as a lipid-driven inflammatory disease; however, this model does not fully explain acute coronary events arising from non-obstructive lesions or the persistence of residual cardiovascular risk despite lipid-lowering therapy. Emerging evidence implicates infectious agents as contributors to plaque instability rather than primary initiators of disease. Epidemiological studies demonstrate temporal associations between infections and myocardial infarction, while molecular analyses have identified bacterial components within atherosclerotic plaques. Recent findings suggest that microorganisms exist within plaques as biofilms, structured communities embedded in an extracellular matrix that permits immune evasion and long-term persistence. Under host-derived physiological triggers, including inflammatory surges and metabolic stress, biofilm dispersion may expose bacterial products that amplify inflammation and contribute to plaque destabilization. This biofilm-centered framework complements the lipid-centric model by identifying microbial factors as amplifiers of plaque vulnerability. It also highlights potential diagnostic and therapeutic targets aimed at reducing residual inflammatory cardiovascular risk.

## Highlights

• Evidence suggests a possible association between chronic infections and atherosclerosis.
• Intraplaque microbial signals may be detected in a subset of atherosclerotic lesions.
• Microbial products may contribute to vascular inflammation and endothelial dysfunction.
• Infection-related pathways could play a role in residual cardiovascular risk.
• Targeting infection-driven inflammation could open new avenues for cardiovascular prevention.

## Graphical abstract

**Figure:**
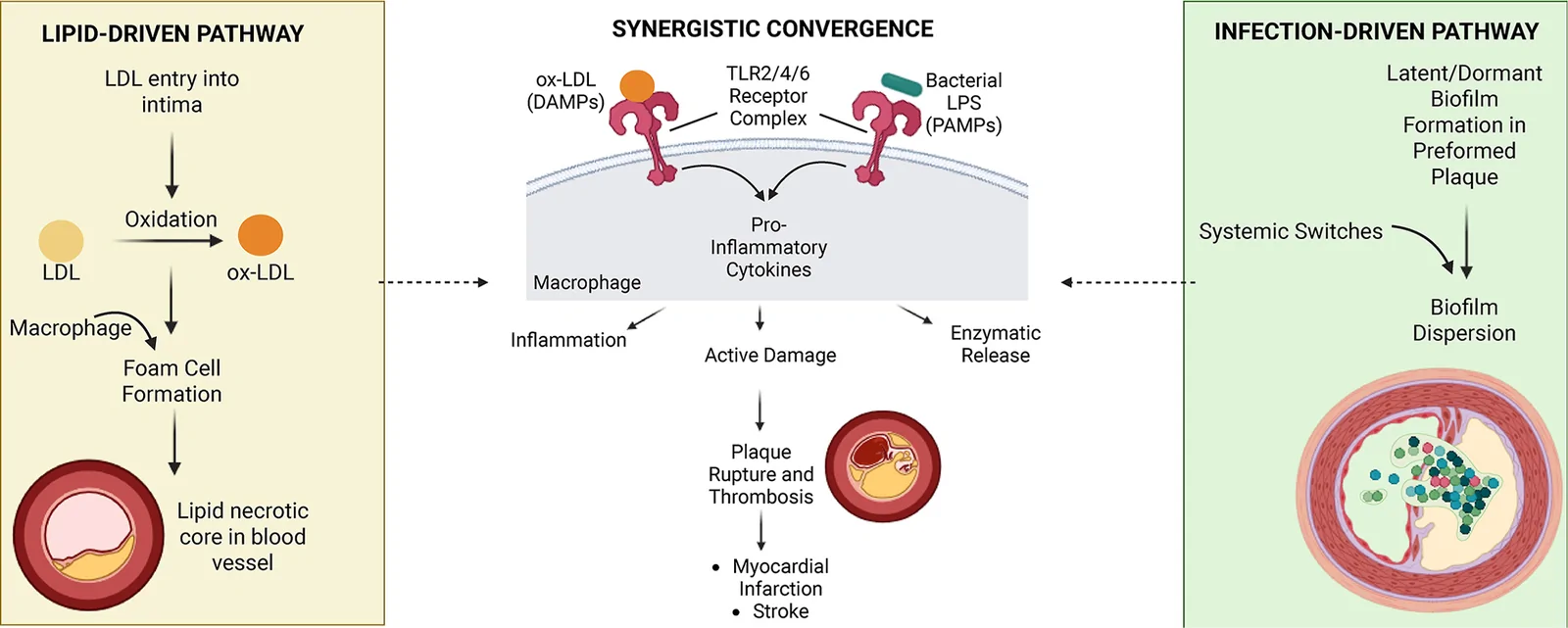

## 1 Introduction

Atherosclerotic plaques are progressive, lipid-driven inflammatory lesions and the cause of coronary artery disease (CAD) and myocardial infarctions (MIs) [1,2]. Over time, the growth of plaques hardens the arteries and narrows the vessel lumen. Acute events occur when plaques rupture, exposing thrombogenic material and triggering clot formation. Importantly, many culprit lesions do not significantly narrow the artery, defined as a stenotic lesion, but instead represent thin-capped, inflamed plaques, emphasizing that plaque composition is more predictive than size [3].

The classical lipid-driven model of atherosclerosis describes endothelial dysfunction leading to low-density lipoprotein (LDL) infiltration, its oxidation (ox-LDL), and activation of innate and adaptive immune responses. Disease progression is driven by chronic inflammation and eventual plaque destabilization when reparative processes fail [4]. Imaging and clinical studies further distinguish between focal and diffuse CAD phenotypes, with focal lesions enriched in lipid-rich plaques and thin-cap fibroatheromas, while diffuse CAD is more often associated with stable calcifications. In addition, focal plaques are more prone to rupture, while diffuse plaques produce gradual ischemia [5].

However, this model does not fully explain why acute events can occur in the absence of severe stenosis or despite well-managed lipid levels. Increasing evidence suggests that infections and pathogen-related inflammation may contribute to plaque instability and act as triggers for acute coronary events, supporting a reevaluation of the infectious hypothesis in CAD [6,7]. Yet, for decades this theory was stalled because large-scale antibiotic trials failed, leading to the assumption that bacteria were merely coincidental bystanders. However, using advanced imaging, bacteria, organized into structured biofilms rather than free-floating, were recently detected in human coronary plaques. This aligns with emerging clinical evidence that microbial biofilms act as silent drivers of chronic systemic inflammatory conditions, including cardiovascular and metabolic diseases [8]. Thus, these bacteria survive immune surveillance and conventional treatment and are capable of triggering sudden plaque ruptures, thereby increasing the risk of plaque destabilization and acute coronary events [9].

## 2 Conventional understanding of atherosclerosis

**Fig. 1:**
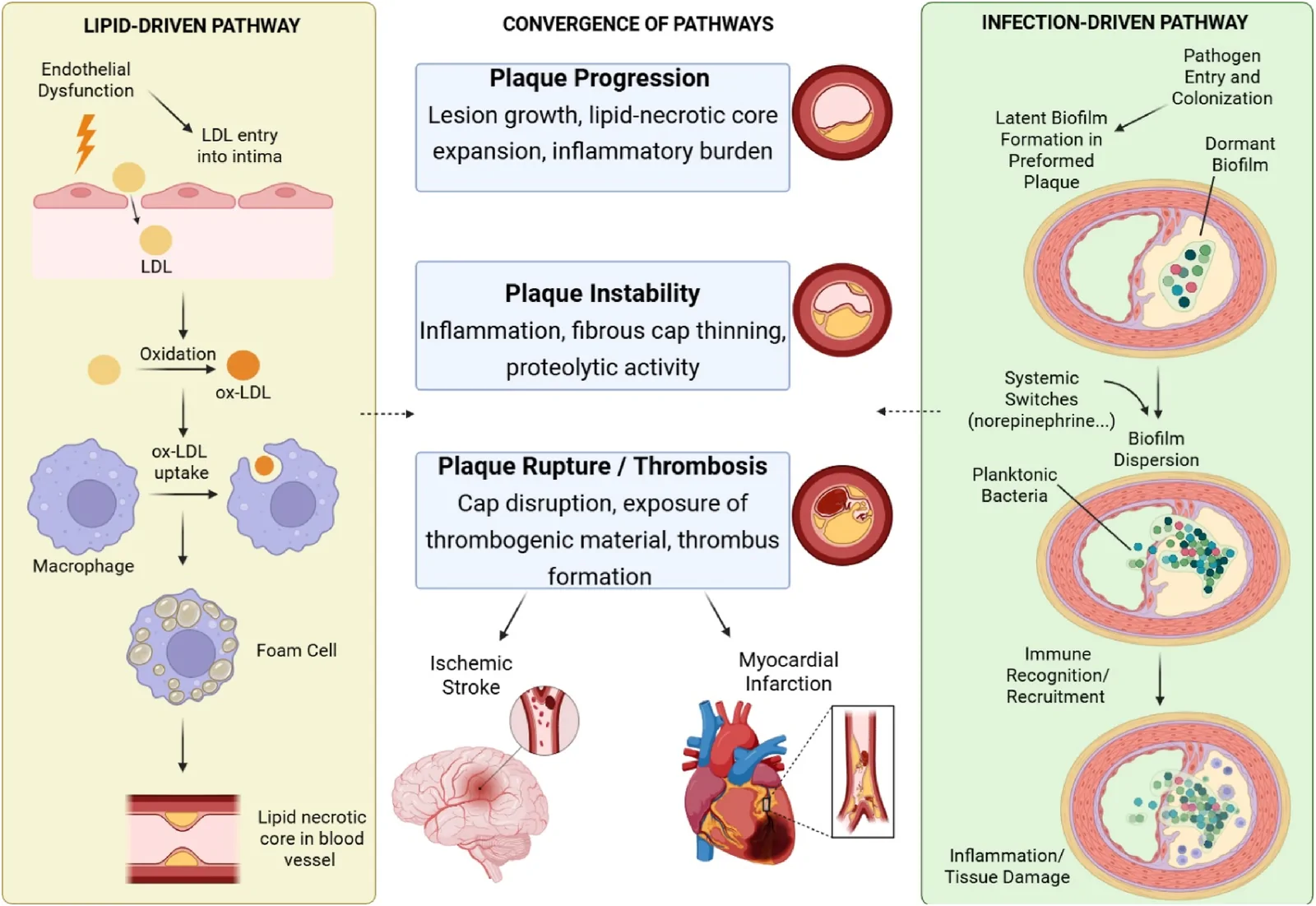
Synergistic Mechanistic Model of Plaque Destabilization via Lipid Accumulation and Latent Biofilm Formation. Left pathway illustrates classic endothelial infiltration of LDL and subsequent foam cell formation. Right pathway depicts the sequestration of pathogens within pre-existing plaque lesions, forming a latent biofilm shield. Environmental switches (norepinephrine or free iron) trigger a phenotype change, leading to a localized biofilm dispersion where planktonic bacteria escape. The convergence of lipid-driven inflammation and biofilm-associated inflammatory activation promotes plaque destabilization and rupture, potentially precipitating acute cardiovascular events such as myocardial infarction and acute coronary syndrome**.** Created in BioRender. El Hayek, C. (2026) https://BioRender.com. LDL: Low-Density Lipoprotein; ox-LDL: Oxidized Low-Density Lipoprotein.

The conventional understanding of atherosclerosis is based on a lipid-driven chronic inflammatory disease of arteries. Endothelial dysfunction initiated by risk factors such as stress, hypertension, and smoking increases vascular permeability, allowing low-density lipoproteins (LDL) to penetrate and accumulate within the arterial intima (Fig. 1). Consequently, trapped LDL undergoes oxidation forming ox-LDL that acts as damage-associated molecular patterns (DAMPs) triggering an inflammatory response [10]. As a result, endothelial cells upregulate adhesion molecules, capturing monocytes. These monocytes migrate into the intima and differentiate into macrophages, which internalize ox-LDL through scavenger receptors, primarily CD36 [11]. This leads to the formation of lipid-laden foam cells that accumulate over time, forming fatty streaks, the earliest visible lesions of atherosclerosis.

**Fig. 2:**
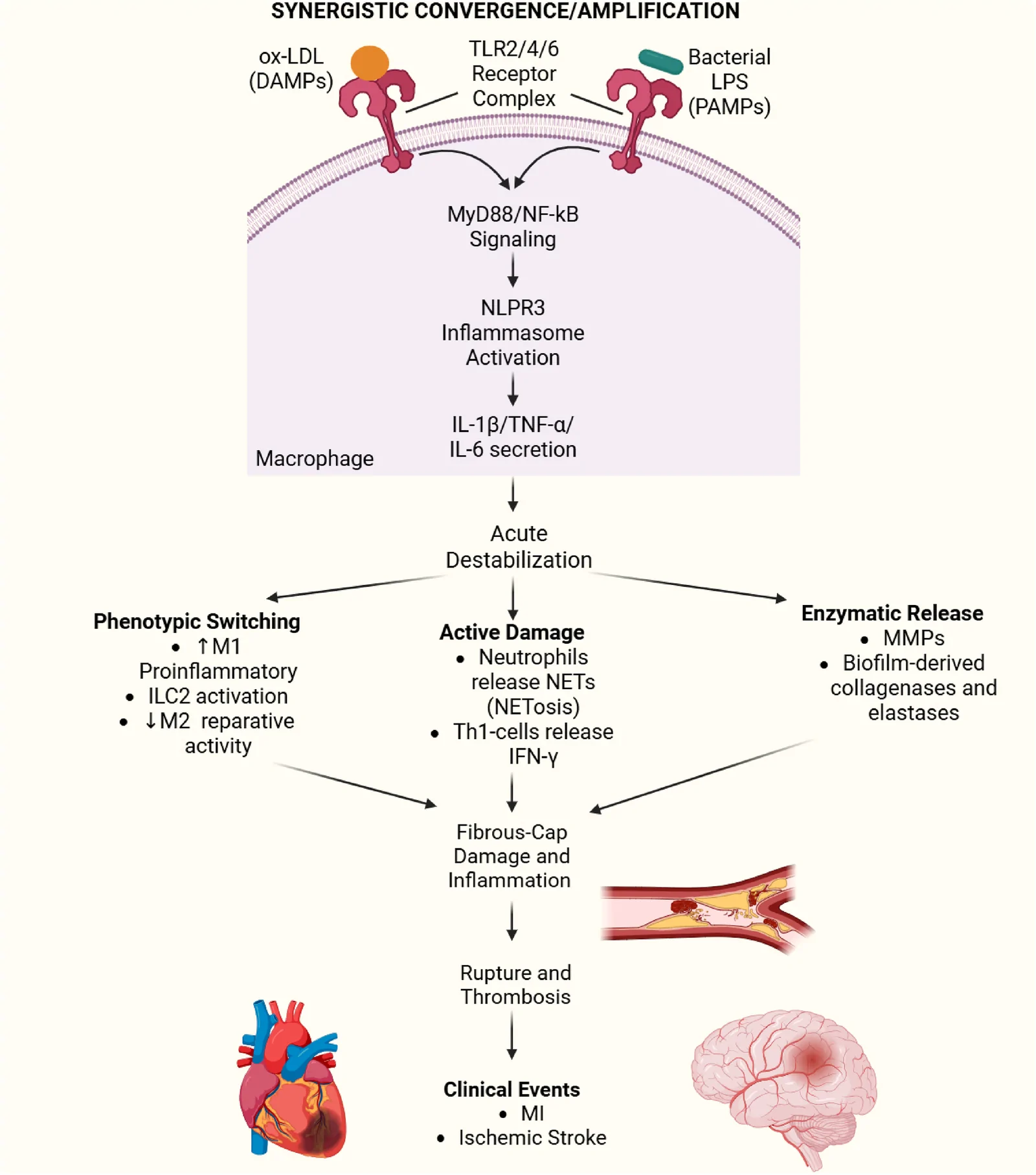
Synergistic convergence of lipid- and infection-derived signals in plaque destabilization. The convergence of DAMPs and PAMPs on macrophage TLR2/4/6 receptors drives downstream MyD88/NF-kB signaling and NLPR3 inflammasome hyperactivation, leading to accelerated macrophage polarization to M1 pro-inflammatory phenotype as well as activation of ILC2 cells, release of NETs from neutrophils and IFN-γ, and enzymatic cap degradation via hist MMPs, resulting in terminal plaque rupture and clinical events. Created in BioRender. El Hayek, C. (2026) https://BioRender.com. DAMPs: Damage-Associated Molecular Patterns; PAMPs: Pathogen-Associated Molecular Patterns; LPS: Lipopolysaccharide; TLR2/4/6: Toll-Like Receptors 2/4/6; MyD88: Myeloid Differentiation Primary Response 88; NF-kB: Nuclear Factor Kappa B; IL-1β: Interleukin-1 Beta; ILC2: Type 2 Innate Lymphoid Cells; M1 Pro-inflammatory Macrophages; M2 Anti-inflammatory Macrophages; IFN-γ: Interferon-Gamma; Th1 Cells: T Helper cells; NETs: Neutrophil Extracellular Traps; MMPs: Matrix Metalloproteinases; MI: Myocardial Infarction.

Foam cells and activated macrophages express pattern recognition receptors, including Toll-like receptors (TLRs), particularly TLR4/TLR6. Recognition and binding of these receptors by ox-LDL transduce the signaling pathway for NF-kB-mediated cytokine production. In return, activated macrophages produce pro-inflammatory cytokines (TNF-α, IL-1β, IL-6), contributing further to the inflammation (Fig. 2) [11].

Disease progression is strongly influenced by macrophage heterogeneity, especially the balance between pro-inflammatory (M1) and anti-inflammatory (M2) phenotypes [12]. Classically, M1 cells are driven by the accumulation of ox-LDL and the secretion of Th1 cytokines to induce plaque vulnerability. On the other hand, M2 macrophages play a protective role by promoting tissue repair and the clearance of apoptotic cells to maintain plaque stability. While this M1/M2 balance is a primary determinant of plaque progression, recent evidence suggests a more complex process where macrophages adopt specialized archetypes [13].

Moreover, this innate response is amplified by adaptive immunity. T-lymphocytes, particularly Th1, secrete IFN-γ, which sustains inflammation and enhances macrophage activation. B-lymphocytes also contribute by producing antibodies against oxidized lipids [14]. Additionally, type 2 innate lymphoid cells (ILC2s) exert an atheroprotective role by releasing cytokines IL-5 and IL-13, which maintain perivascular adipose tissue homeostasis and limit plaque progression [15,16]. Neutrophils contribute to plaque vulnerability by releasing neutrophil extracellular traps (NETs) that drive both the local inflammatory response and subsequent thrombotic events. Thus, distinct immune cell behavior of the innate and adaptive response, where certain cells act as atheroprotective while others are pro-atherogenic, allow the transition from early fatty streaks to advanced lesions. While monocytes and macrophages dominate the early stages, the later stages are characterized by defective clearing of dying cells. This failure, combined with T-cell-mediated thinning of the fibrous cap creates a necrotic environment that predisposes the plaque to rupture [17].

As lesions evolve, vascular smooth muscle cells migrate from the media into the intima, where they undergo phenotypic switching and secrete collagen to form a fibrous cap covering the lipid-rich necrotic core. However, persistent inflammation promotes the degradation of the fibrous cap through the activity of matrix metalloproteinases and elastases, resulting in cap thinning. Consequently, thin cap fibroatheromas are highly vulnerable and susceptible to rupture under stress. Upon rupture, the highly thrombogenic necrotic core is exposed to the bloodstream triggering platelet activation and thrombus formation. This process underlies acute clinical events such as myocardial infarction (MI) and stroke [18]. In advanced fibroatheromas, the accumulation of these innate cells and the decreased ration of protective T-regulatory cells create an environment in which the degradation of the fibrous cap by matrix metalloproteinases is significantly accelerated [17].

## 3 Clinical observations unexplained by the conventional model

While the conventional model successfully explains many aspects of coronary artery disease, several clinical observations remain insufficiently addressed. Despite advances in lipid-lowering therapies, patients still suffer from many cardiovascular events, a phenomenon referred to as residual risk [19]. Clinical trials of statins have drastically lowered LDL levels and event rate; however, many patients still suffer from MI or stroke. This suggests that non-lipid factors contribute to the pathogenesis.

The mechanisms that convert a stable plaque into an unstable rupture-prone lesion are not well understood, and the timing of acute events is frequently linked to external triggers. Epidemiological studies have shown that infections such as those involving respiratory viruses can act as potent triggers for MI [20]. In a seminal study using large-scale primary care data, it was observed that the risk for both MI and stroke is elevated in the first three days following a respiratory diagnosis [21]. This association was further reinforced by using laboratory-confirmed influenza cases, which showed an increased risk of MI during the first week of infection [22]. Additionally, influenza vaccination was associated with a reduction in MI and mortality in CAD patients [23]. Moreover, the presence of non-obstructive CAD in patients who present with MI further indicates that the severity of stenosis is a poor predictor for plaque rupture [24]. This shifts the focus to an acute inflammatory stimulus or the biological composition of the lesion, rather than its size, in destabilizing the plaques [25]. While epidemiological data support a temporal association between acute infections and cardiovascular events, they also raise the possibility that infectious agents may play a more direct role in atherosclerotic disease mechanisms.

## 4 Infections in atherosclerosis and pathogens implicated

The hypothesis that infectious agents contribute to the pathogenesis of atherosclerosis gained traction in the early 20th century, when it was first proposed that toxic agents could induce arterial hardening. Modern validation emerged later when Marek's disease herpesvirus was shown to induce atherosclerotic lesions in chickens, independent of cholesterol status [26]. Subsequent human studies strengthened this concept. Seroepidemiological evidence demonstrated a higher prevalence of *Chlamydia pneumonia* (*C.* pneumonia) in CAD patients (Table 1) [27], while molecular studies identified Cytomegalovirus DNA within human arterial walls [28]. These foundational studies provided early motivation to consider infection as a co-factor in atherogenesis rather than a coincidental association.

**Table 1:** Important pathogens implicated in atherosclerosis.

Pathogen	Route of Entry	Mechanism of Action	References
*Porphyromonas gingivalis* (oral)	Enters bloodstream via chewing, brushing, or dental procedures	• Invades endothelial cells • Accelerates foam cell formation • Alters lipid metabolism in vitro • Transitions to a VBNC state	Haraszthy et al., 2000; Kozarov et al., 2005; Qi et al., 2003
Chlamydia Pneumonia (respiratory)	Disseminates from the lungs via infected macrophages	• Leads to diffuse atherosclerotic changes • Triggers chronic low-grade vascular inflammation	Saikku et al., 1988; Legan et al., 2004
Viridans Streptococci	Migrates from dental biofilm to vascular tissue	• Organizes into biofilms within the EPS matrix • Leads to advanced lesions and associated with rupture-prone fibroatheromas	Karhunen et al., 2025
Staphyloccous spp., Proteus spp, Klebsiella spp.	Hematogenous spread likely from skin, gut, or urinary tract	• Contributes to bacterial diversity within the lesion • Supports intraplaque microbiome stability	Ott et al., 2006

EPS: Extracellular polymeric substance; VBNC: viable but non-culturable.

Interest subsequently expanded toward chronic inflammatory conditions as sustained sources of vascular insult. In this context, periodontal disease has emerged as a significant cardiovascular risk factor [29,30]. Early epidemiological data first established a clinical association between poor dental health and acute MI [31], a relationship later supported by mechanistic evidence. *Porphyromonas gingivalis* (*P. gingivalis*), a key periodontal pathogen, was identified in atherosclerotic plaques using PCR and immunohistochemistry (Table 1) [32,33], where it can accelerate foam cell formation and alter lipid metabolism in vitro [34]. Oral bacteria can enter the bloodstream during routine activities such as chewing and tooth brushing, leading to transient bacteremia. Once in circulation, these microorganisms can adhere to endothelial cells, invade vascular tissues, and modulate local immune responses. For example, hematogenous dissemination of oral periodontopathogens, such as *P. gingivalis*, directly compromises endothelial cell integrity, specifically through gingipain-mediated cleavage of vascular endothelial cadherins and upregulated expression of chemo attractants, such as monocyte chemotactic protein (MCP)-1. This, in turn, accelerates monocyte recruitment and subendothelial oxidation [35].

Further, some studies suggest that specific pathogens may drive a more aggressive, diffuse form of coronary artery disease. Histopathological studies have identified a significant association between chronic *C*. *pneumoniae* infection and diffuse atherosclerosis changes [36]. In addition, analyses of human vascular samples identified diverse bacterial populations with an average bacterial diversity score of 12.33 ± 3.81 distinct bacterial phylotypes per plaque sample with a range of 5 to 22 [37], supporting the emerging concept of an intraplaque microbiome rather than the presence of a single dominant pathogen. Using conserved PCR, the study found that bacterial DNA was found in all CHD patients, while none was detected in healthy control coronary arteries. The most prevalent organisms included Staphylococcus species, *Proteus vulgaris*, *Klebsiella pneumoniae*, *Streptococcus* species as well as *Chlamydia* species (Table 1).

In addition to direct vascular colonization by microorganisms, evidence supports indirect mechanisms linking infection and cardiovascular risk. An oral–gut–atherosclerosis axis has been proposed, whereby periodontal pathogens are associated with alterations in intestinal microbiota and accelerated atherosclerotic disease progression [38]. Experimental periodontitis in ApoE^−/−^ mice induced gut dysbiosis and an increase in circulating trimethylamine N-oxide (TMAO), a gut microbiota–derived metabolite of dietary choline and carnitine [39,40]. TMAO has been implicated in atherosclerosis through multiple mechanisms, including impaired reverse cholesterol transport, promotion of macrophage foam cell formation via increased scavenger receptor expression, and activation of pro-inflammatory signaling pathways [[41], [42], [43], [44]]. Thus, TMAO represents a key downstream mediator linking microbial activity to host cardiovascular risk.

## 5 Biofilm-mediated persistence of bacteria in atherosclerosis

Despite accumulating evidence linking infection to atherosclerosis through both local vascular involvement and systemic metabolic pathways, a key open question is how microorganisms persist within atherosclerotic lesions despite antimicrobial exposure and immune surveillance. For decades, the infectious hypothesis of atherosclerosis was supported by the consistent detection of bacterial DNA and antigens within human plaques. For this reason, several trials such as WIZARD and ACES [45,46] tested whether administering broad-spectrum antibiotics for the suspected microorganisms to CAD patients could slow atherosclerosis. However, these trials showed no significant reduction in cardiovascular events. These results weakened the possible role of infections and led many to conclude that microbial involvement was not clinically meaningful.

One major methodological constraint of this approach was the inability to distinguish between association and causation. It was not understood whether bacteria initiate plaque formation, actively contribute to disease progression, or simply colonize an already diseased tissue [6]. However, retrospective analysis suggests that these trials may be limited by an incomplete understanding of bacterial persistence, survival, and antibiotic resistance mechanisms. The trials were designed under the assumption that bacteria existed as planktonic, metabolically active, and susceptible to antibiotics. Further, emerging evidence indicates that vascular pathogens survive under host-induced oxidative stress by transitioning into a viable but non-culturable (VBNC) state (Table 1). In this dormant state, bacteria such as *P. gingivalis* retain their capacity for intracellular invasion of endothelial cells despite remaining undetectable by routine culture techniques, providing a mechanism for long-term vascular persistence, immune evasion, and tolerance to standard antibiotics [47].

Emerging evidence challenges this assumption by demonstrating that bacteria within atherosclerotic plaques can adopt a biofilm phenotype. With fluorescence in situ hybridization and confocal microscopy, bacteria within plaques were noted to be organized within an extracellular polymeric substance (EPS) matrix [48]. This allows biofilm formation where the EPS acts as a physical barrier that restricts antibiotic diffusion, while the microenvironment within induces a state of metabolic quiescence evading immune detection and persisting for prolonged periods of time [49].

More recent studies support this concept by identifying viridans streptococci, a common constituent of the oral microbiota, within human coronary plaques (Table 1). More importantly, the bacteria were not randomly distributed but rather organized into biofilms shielded from immune detection. Moreover, plaque immunostaining shows streptococci more often in advanced lesions, especially in advanced fibroatheromas and rupture-prone lesions [9].

## 6 Mechanisms linking infection to plaque destabilization

Biofilms are increasingly implicated in plaque destabilization, especially in the shift from stable to rupture-prone plaques as they trigger an acute inflammatory response that destabilizes the plaque. In advanced but stable plaques, bacteria may persist in a dormant biofilm state, sequestered within the lesion and evading macrophage detection (Fig. 1). In contrast, rupture-prone plaques show evidence of bacterial infiltration into the fibrous cap, where they trigger localized innate and adaptive immune responses [9]. This suggests that systemic immune activation could potentially disrupt biofilm integrity exposing bacterial components to the host immune system activating the innate immune receptors such as TLR2 and TLR4 and downstream Myd88-NF-Kb signaling, with amplification of pro-inflammatory cytokine release. Additionally, bacterial pathogen-associated molecular patterns (PAMPs) such as LPS and fimbriae are potent activators of NETosis, the process of induction of NETs, through TLR4 mediated signaling, leading to plaque instability [50]. Adaptive immune responses, specifically T-cells appear secondary compared to the robust innate response. Moreover, while type 2 innate lymphoid cells (ILC2s) typically provide protective role by responding to pathogen-induced tissue injury with reparative cytokines, severe systemic infections can dysregulate this innate response, potentially shifting ILC2s toward an inflammatory phenotype that could exacerbate plaque instability (Fig. 2) [16,51].

In parallel, biofilm dispersion itself may directly weaken plaque structure. In vitro studies show that plaque-derived biofilms can release matrix-degrading enzymes, such as collagenases, upon transitioning to a planktonic state. This dispersion can be triggered by physiological cues such as elevated free iron levels, a common byproduct of intraplaque hemorrhage [52]. Moreover, biofilms can polarize macrophages toward the pro-inflammatory M1 phenotype while simultaneously suppressing the anti-inflammatory M2 phenotype, which not only exacerbates local inflammation but also impairs the structural integrity of the fibrous cap by reducing collagen production (Fig. 2) [53].

These mechanisms link biofilm persistence and activation to plaque destabilization and rupture. Rather than acting as primary drivers of atherosclerosis, bacteria may function as latent amplifiers that destabilize established plaques. Under conditions such as systemic inflammation, iron overload or catecholamine stimulation (norepinephrine), biofilm disruption may occur, leading to bacterial exposure and secondary immune activation that amplifies inflammatory injury and promotes plaque rupture [48,52].

## 7 Interplay between lipid-driven atherosclerosis and infectious inflammation

Infectious mechanisms of plaque destabilization integrate with, rather than replace the lipid-centric model of atherosclerosis. It may also help resolve a long-standing paradox in clinical cardiology: the occurrence of fatal MI in patients with only mild to moderate coronary stenosis. Bacterial processes appear to be more closely associated with plaque instability and rupture than with plaque size and degree of obstruction, suggesting that even non-obstructive lesions may be vulnerable to biofilm-driven destabilization [9]. However, lipid accumulation and chronic inflammation remain essential for plaque formation.

This integrated model can further help explain how the residual risk can operate independently of lipid levels. Statins can reduce but not eliminate cardiovascular risk, indicating the contribution of non-lipid inflammatory pathways. Although the trial did not explicitly investigate microbial triggers, the CANTOS trial has shown that targeting IL-1β significantly reduced cardiovascular events independent of lipid levels [54]. On the other hand, chronic bacterial stimulation through lipopolysaccharide can activate NLPR3 inflammasome within macrophages and endothelial cells [55,56]. Those two pathways intersect since NLPR3 can then activate the IL1-β pathway among others [57]. Thus, the atherosclerotic environment contains both exogenous PAMPs and endogenous DAMPs that utilize the same TLR/NF-kB signaling pathway (Fig. 2). This hyper-activates the NLPR3 inflammasome, explaining why the presence of low-grade infection can significantly amplify the inflammatory response to lipids even when lipids levels are medically controlled [50]. These mechanisms provide a potential link between lipid accumulation, chronic inflammation, and infection-driven immune activation in plaque destabilization.

## 8 Clinical implications

These mechanistic insights raise important implications for the development of more specific clinical biomarkers and risk-stratification strategies. From a clinical perspective, traditional biomarkers such as C-reactive protein and IL-6 reflect systemic inflammation and were associated with major adverse cardiovascular events and mortality [58]. However, these markers lack specificity for identifying localized plaque vulnerability [59]. Similarly, conventional diagnostic tools such as cardiac stress test and coronary angiography primarily detect significant stenoses, yet a majority of coronary events result from non-obstructive lesions that evade detection methods [3]. Thus, developing diagnostic modalities that can assess the biological status of the arterial wall could allow the identification of high-risk patients who appear healthy under conventional screening.

Novel biomarkers reflecting infection-associated vascular activity may offer additional clinical value. For example, circulating biofilm-specific antibody titers or detection of bacterial genetic material could serve as early indicators for plaque destabilization. Specifically, research has linked the presence of bacterial genes such as *wcaD* and *sdhC* from Enterbacterales to elevated levels of host antimicrobial peptide LL-37 and pro-inflammatory metabolite 12S-HETE. Such markers may help identify high-risk patients who might otherwise be classified as low-risk based on traditional lipid profiles [60].

Moreover, oral bacteria, which are among the most commonly implicated pathogens, highlight the potential importance of maintaining good dental health in reducing cardiovascular disease risk. In fact, epidemiological data already link periodontal treatment to reduced systemic inflammatory markers [61].

## 9 Future directions and conclusion

While the presence of bacterial biofilms within plaques is significant, causality remains unproven. Many studies rely on cross-sectional analysis of plaques obtained postmortem or during surgery, limiting the ability to infer temporal relationships. Prospective longitudinal studies are therefore required to determine whether biofilm-positive plaques are predictive of future cardiovascular events, and to define whether biofilms are present in all patients or are restricted to a specific subpopulation. Host genetic susceptibility, immune competence, and overall microbial burden are likely to influence individual risk. In addition, the distribution of biofilms across different CAD phenotypes has yet to be established.

Mechanistically, intraplaque biofilms preferentially sequester within lipid-rich necrotic cores, where they contribute to chronic inflammation and fibrous cap thinning. However, it remains unclear how biofilm dynamics are regulated within the plaque microenvironment, particularly with respect to transitions between sessile to dispersed state that may precipitate plaque rupture.

Future research should also focus on longitudinal studies to assess if positive biofilm plaques are more likely to rupture and cause clinical events as well as investigate whether targeted antimicrobial, biofilm disrupting, or immunomodulatory therapies can complement existing lipid-lowering and anti-thrombotic strategies.

In conclusion, current evidence supports a model in which microbial biofilms may act as modulators of plaque inflammation and instability within established atherosclerotic lesions. This help explain why some plaques rupture spontaneously and why traditional control of risk factors does not fully eliminate cardiovascular events. Rather than replacing lipid-centric paradigm, it integrates infection and immunity as additional determinants of plaque behavior. Comprehensive understanding of these interactions may improve risk stratification and support development of adjunctive therapeutic strategies for the prevention of MI and strokes.

## Author contributions

Conceptualization, C.H., F.K, G.A, G.W.B, and F.A.Z.; Validation, G.W.B., and F.A.Z.; Resources, G.W.B, and F.A.Z.; Data Curation, G.W.B., and F.A.Z.; Writing – Original Draft Preparation, C.H., F.K., G.A., G.W.B, and F.A.Z.; Writing – Review & Editing, C.H., F.K., G.A., G.W.B, and F.A.Z.; Visualization, G.W.B, and F.A.Z.; Supervision, C.H., F.K., G.A., G.W.B, and F.A.Z.; Project Administration, C.H., F.K., G.A., G.W.B, and F.A.Z.; Funding Acquisition, C.H., F.K., G.W.B., and F.A.Z.

## Sources of funding

This work was supported by grants from the 10.13039/100007688American University of Beirut and the Faculty of Medicine: to FAZ [University Research Board (10.13039/501100016263URB)–103949; URB–104262; URB– 104115; URB–104524; Medical Practice Pan (MPP)–320145]. FK would like to acknowledge the support of the Scholarly Concentration Track (10.13039/100027875SCT) at the 10.13039/100019603Faculty of Medicine at the American University of Beirut.

## Declaration of competing interests

The authors declare that they have no known competing financial interests or personal relationships that could have appeared to influence the work reported in this paper.
